# Intravenous fluid resuscitation is associated with septic endothelial glycocalyx degradation

**DOI:** 10.1186/s13054-019-2534-2

**Published:** 2019-07-23

**Authors:** Joseph A. Hippensteel, Ryo Uchimido, Patrick D. Tyler, Ryan C. Burke, Xiaorui Han, Fuming Zhang, Sarah A. McMurtry, James F. Colbert, Christopher J. Lindsell, Derek C. Angus, John A. Kellum, Donald M. Yealy, Robert J. Linhardt, Nathan I. Shapiro, Eric P. Schmidt

**Affiliations:** 1 Department of Medicine, University of Colorado Denver, Aurora, CO USA; 2 Department of Emergency Medicine, Beth Israel Deaconess Medical Center, Boston, MA USA; 3 Departments of Chemistry and Chemical Biology, Chemical and Biological Engineering, and Biomedical Engineering, Rensselaer Polytechnic Institute, Troy, NY USA; 4 Department of Biostatistics, Vanderbilt University Medical Center, Nashville, TN USA; 5 Department of Critical Care Medicine, University of Pittsburgh, Pittsburgh, PA USA; 6 Department of Emergency Medicine, University of Pittsburgh, Pittsburgh, PA USA; 7 Department of Medicine, Denver Health Medical Center, Denver, CO USA

**Keywords:** Sepsis, Multiple organ failure, Endothelial glycocalyx, Fluid resuscitation

## Abstract

**Background:**

Intravenous fluids, an essential component of sepsis resuscitation, may paradoxically worsen outcomes by exacerbating endothelial injury. Preclinical models suggest that fluid resuscitation degrades the endothelial glycocalyx, a heparan sulfate-enriched structure necessary for vascular homeostasis. We hypothesized that endothelial glycocalyx degradation is associated with the volume of intravenous fluids administered during early sepsis resuscitation.

**Methods:**

We used mass spectrometry to measure plasma heparan sulfate (a highly sensitive and specific index of systemic endothelial glycocalyx degradation) after 6 h of intravenous fluids in 56 septic shock patients, at presentation and after 24 h of intravenous fluids in 100 sepsis patients, and in two groups of non-infected patients. We compared plasma heparan sulfate concentrations between sepsis and non-sepsis patients, as well as between sepsis survivors and sepsis non-survivors. We used multivariable linear regression to model the association between volume of intravenous fluids and changes in plasma heparan sulfate.

**Results:**

Consistent with previous studies, median plasma heparan sulfate was elevated in septic shock patients (118 [IQR, 113–341] ng/ml 6 h after presentation) compared to non-infected controls (61 [45–79] ng/ml), as well as in a second cohort of sepsis patients (283 [155–584] ng/ml) at emergency department presentation) compared to controls (177 [144–262] ng/ml). In the larger sepsis cohort, heparan sulfate predicted in-hospital mortality. In both cohorts, multivariable linear regression adjusting for age and severity of illness demonstrated a significant association between volume of intravenous fluids administered during resuscitation and plasma heparan sulfate. In the second cohort, independent of disease severity and age, each 1 l of intravenous fluids administered was associated with a 200 ng/ml increase in circulating heparan sulfate (*p* = 0.006) at 24 h after enrollment.

**Conclusions:**

Glycocalyx degradation occurs in sepsis and septic shock and is associated with in-hospital mortality. The volume of intravenous fluids administered during sepsis resuscitation is independently associated with the degree of glycocalyx degradation. These findings suggest a potential mechanism by which intravenous fluid resuscitation strategies may induce iatrogenic endothelial injury.

## Background

Since its introduction during the cholera epidemics of the nineteenth century, intravenous fluid resuscitation has served as a mainstay of supportive sepsis care [1, 2]. Today, there is increasing concern that intravenous fluids may unexpectedly augment septic endothelial dysfunction, potentially negating the beneficial hemodynamic effects of fluid resuscitation [3]. Such iatrogenic injury could explain the findings of several recent randomized trials which demonstrated that early bolus intravenous fluids worsened sepsis survival [4, 5], as well as observational studies that identified associations between fluid administration [6, 7], fluid balance [8–12], and adverse outcomes.

**Fig. 1:**
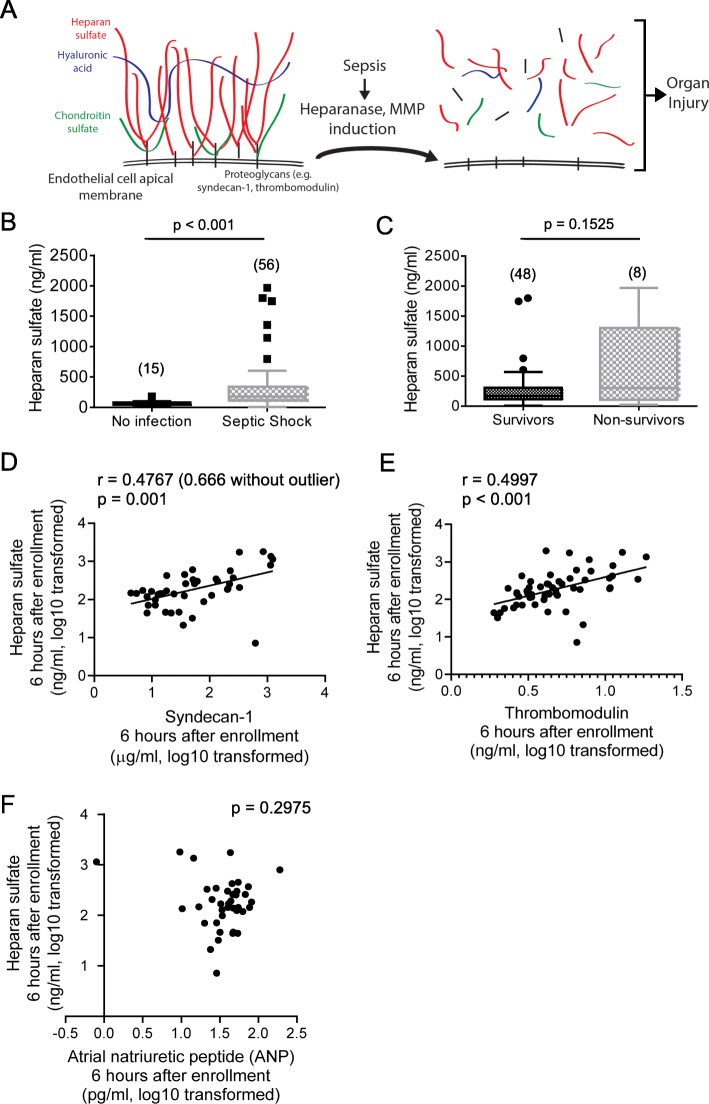
Glycocalyx degradation occurs in patients with septic shock. **a** The endothelial glycocalyx is an apical endothelial layer composed of transmembrane proteoglycans (such as syndecan-1 and thrombomodulin) covalently attached to glycosaminoglycans (primarily heparan sulfate) that project into the vascular lumen. During sepsis, activation of heparanase and matrix metalloproteinases leads to glycocalyx degradation, releasing heparan sulfate and proteoglycan fragments into the plasma. **b** In a cohort of 56 septic shock patients enrolled in the ProCESS trial, circulating heparan sulfate levels were elevated in comparison to 15 non-infected ED controls. Measurements in septic patients were made 6 h after enrollment (i.e., after initial fluid resuscitation). **c** Of 56 septic patients, 8 patients eventually died during their hospitalization. There was a non-significant trend towards increased heparan sulfate concentrations (measured after 6 h resuscitation) in non-survivors. Circulating heparan sulfate concentrations (at 6 h) correlated with plasma concentrations of glycocalyx components syndecan-1 (**d**) and thrombomodulin (**e**) in septic shock patients. Line represents best fit line. **f** There was no association between plasma heparan sulfate and atrial natriuretic peptide (ANP) in septic shock patients after 6 h resuscitation. Parentheses in **b**, **c** represent number of patients in each group

The mechanisms by which intravenous fluid resuscitation could cause harm are uncertain. Preclinical studies suggest that intravenous crystalloids promote degradation of the endothelial glycocalyx [13], a ubiquitous endothelial cell-surface layer composed of transmembrane or membrane-anchored proteoglycans (such as syndecan-1) covalently decorated with sulfated glycosaminoglycans (predominantly heparan sulfate, Fig. 1a). The glycocalyx is essential to microvascular homeostasis, as it contributes to the endothelial barrier, mediates shear-induced vasorelaxation, and opposes leukocyte-endothelial adhesion [14]. During sepsis, tumor necrosis factor-α [15] and angiopoietin-2 [16] induce endothelial expression and activation of heparanase, an endoglucuronidase that degrades glycocalyx heparan sulfate, inducing endothelial dysfunction and consequent organ injury. Heparanase and inflammatory stimuli may additionally induce metalloproteinase-mediated shedding of syndecan-1 [17], potentially augmenting glycocalyx collapse. Therefore, the presence of circulating glycocalyx constituents such as heparan sulfate or syndecan-1 fragments indicates a loss of glycocalyx integrity and associated endothelial injury [18].

Preclinical studies have suggested that atrial natriuretic peptide (ANP), a hormone released in response to volume loading-induced atrial stretch, is sufficient to degrade the endothelial glycocalyx in non-septic animals and humans [19–21]. Similarly, a preclinical study of ovine endotoxemia observed that intravenous fluid resuscitation induced a simultaneous rise in circulating glycocalyx fragments and plasma ANP, coincident with worsened septic vasoplegia [13]. These concordant observations suggest that ANP upregulation could be a mechanism for volume overload-related glycocalyx degradation independent of tumor necrosis factor-α and angiopoietin-2-related degradation. The association between ANP and glycocalyx degradation in septic humans, however, has not been explored.

To explore the potential importance of the glycocalyx in human sepsis pathophysiology as well as the association between intravenous fluid resuscitation and glycocalyx degradation, we measured circulating glycocalyx constituents in (a) a subgroup of septic shock patients enrolled in the Protocolized Care for Early Septic Shock (ProCESS) trial and (b) sepsis patients presenting to emergency departments (EDs) at the Beth Israel Deaconess Medical Center (BIDMC, Boston, MA, USA) or St. Vincent’s Hospital (Worchester, MA, USA). We hypothesized that (1) the degree of glycocalyx degradation, as measured by circulating heparan sulfate, is associated with sepsis severity and mortality and (2) the volume of intravenous fluids administered early in resuscitation is independently associated with the degree of glycocalyx degradation.

## Methods

### Study populations

The study population for the ProCESS study [22] and the ProCESS Microcirculatory Flow Ancillary Study [23] has been described in detail elsewhere. In brief, subjects all had (a) suspected infection in the ED, (b) at least two systemic inflammatory response syndrome (SIRS) criteria [24], and (c) refractory hypotension defined as a systolic blood pressure < 90 mmHg despite an IV fluid challenge of at least 1 l crystalloids or evidence of tissue hypoperfusion (blood lactate concentration ≥ 4 mmol/l). We used a convenience sample of 56 patients enrolled at sites participating in the ProCESS Microcirculatory Flow Ancillary Study [23] to perform an initial assessment of glycocalyx degradation in sepsis. We measured plasma heparan sulfate in samples collected 6 h after ProCESS study enrollment, coinciding with the completion of initial volume resuscitation. For comparison, we performed measurements using samples collected from 15 patients presenting with minor, non-infectious complaints to EDs at either BIDMC, Massachusetts General Hospital, or Brigham and Women’s Hospital (Boston, MA, USA). In ProCESS patients, we additionally measured levels of circulating syndecan-1 as a second marker of glycocalyx degradation to determine if a more inexpensive ELISA-based assessment of glycocalyx degradation correlated with the “gold standard” of circulating heparan sulfate levels by mass spectrometry.

To validate observations in the ProCESS patients, we enrolled a second group of patients recruited from BIDMC and St. Vincent’s Hospital. Patients were adult (age > 18 years) ED patients presenting with suspected sepsis, enrolled on a convenience basis. We included patients representative of the entire spectrum of sepsis severity, defined by maximum sepsis syndrome severity in the first 72 h of study enrollment [25]. We selected 100 subjects split roughly evenly between sepsis severities: infected/sepsis patients, severe sepsis (sepsis plus organ dysfunction), and septic shock (sepsis plus systolic blood pressure < 90 mmHg after a minimum of 1 l intravenous fluid administration) to comprise our study cohort. Thirty ED patients presenting with minor non-infectious complaints served as controls. In this cohort, we collected samples from patients at ED presentation and 24 h later, and we recorded the volumes of intravenous fluids administered between these time points. All samples were processed within 60 min of being obtained and stored at − 80 °C until analysis.

The University of Pittsburgh and BIDMC Committees for Clinical Investigations, and the local review boards at each enrolling site approved the study design. Each subject or legal authorized representative gave written informed consent.

### Antibodies and reagents

We measured plasma syndecan-1 (ab46506, Abcam, Cambridge, MA, USA) and brain natriuretic peptide (BNP; ab193694, Abcam) by ELISA, and plasma ANP (RAB0385, Millipore Sigma, St. Louis, MO, USA) by EIA. For the ProCESS cohort, indices of endothelial injury and coagulation including thrombomodulin (an endothelial surface chondroitin sulfate glycosaminoglycan), soluble fms-like tyrosine kinase (sFLT-1; also known as soluble vascular endothelial growth factor receptor-1), angiopoietin 2, and tissue plasminogen activator (tPA) were measured as previously described [26]. Serum lactate and D-dimer were measured as part of the parent ProCESS study. Serum interleukin 6 and tumor necrosis factor-α were measured by ELISA (Quantikine, R&D Systems, Minneapolis, MN, USA).

### Quantification of plasma heparan sulfate

As previously described, we isolated glycosaminoglycans from EDTA plasma using a spin-column approach [27]. After desalting, we enzymatically digested glycosaminoglycans into component disaccharides. We then 2-aminoacridone-labeled disaccharides and quantified heparan sulfate concentrations using liquid chromatography-mass spectrometry multiple reaction monitoring (LC-MS/MS MRM) [28]. This highly sensitive approach, previously developed [29] and validated [30] by our group, is capable of detecting circulating heparan sulfate of all sulfation types, contrasting the limitation of antibody-based assays to only a few sulfation patterns. We have previously demonstrated that this LC-MS/MS MRM approach to measuring circulating heparan sulfate is highly sensitive to both septic and non-septic glycocalyx degradation [31] and is an early predictor of glycocalyx degradation-associated organ injury [28].

### Statistical analysis

For the ProCESS microcirculatory flow cohort, we used samples available from the 6-h (post-resuscitation) timepoint to assess differences in levels of circulating heparan sulfate between patients with and without sepsis, and between survivors and non-survivors. We used linear regression to evaluate the association between intravenous fluid volume and heparan sulfate levels, adjusting for age and severity of illness using the Sequential Organ Failure Assessment (SOFA) score at presentation. We considered variables such as demographics (e.g., age, gender) and co-morbidities (e.g., congestive heart failure and chronic kidney disease) for the model and used a forward selection model, allowing variables below the threshold of *p* < 0.2 to be eligible to enter the model, and retaining covariates significant at the *p* < 0.05 threshold.

We repeated this approach for the BIDMC/St. Vincent’s cohort, comparing heparan sulfate levels at ED presentation with maximum sepsis syndrome severity within 72 h. Additionally, we assessed the relationship between the volume of intravenous fluids administered in the 24 h following ED presentation and the change in circulating heparan sulfate over this time period, using a linear regression model adjusted for age and severity of illness (baseline SOFA score). We repeated the analysis stratified by sepsis syndrome at presentation to further assess the relationship with illness severity.

For both cohorts, we analyzed data using Prism (GraphPad, San Diego, CA, USA) and SAS (Cary, NC, USA) for multivariable analyses. For comparison of two groups, we used a Mann-Whitney test. For comparison of multiple groups, we used Kruskal-Wallis testing with Dunn’s post hoc analysis for two-group comparisons. We assessed correlations by Pearson’s correlation of log-transformed data. We performed receiver-operating characteristic curves for in-hospital mortality. We share data as box and whisker graphs (demonstrating median, 25th, and 75th percentile data with Tukey representation of outliers) and set the per-comparison alpha error at 0.05.

## Results

### ProCESS patient cohort

Analyses of plasma samples collected from ProCESS patients (“ProCESS Study”, Table 1) after completion of trial-directed fluid resuscitation (6 h after patient enrollment) demonstrated higher levels of circulating heparan sulfate as compared to non-infected ED controls (Fig. 1b). This elevation of circulating heparan sulfate in septic patients is consistent with previous reports [27, 32]. There was a non-statistically significant trend towards increased plasma heparan sulfate in the 8 patients of this cohort who died later in their hospitalization (Fig. 1c), with an area under the ROC curve of 0.661 (*p* = 0.1466). Plasma heparan sulfate correlated with other measures of glycocalyx degradation, such as the shed proteoglycans syndecan-1 (Fig. 1d) and thrombomodulin (Fig. 1e).

**Table 1:**
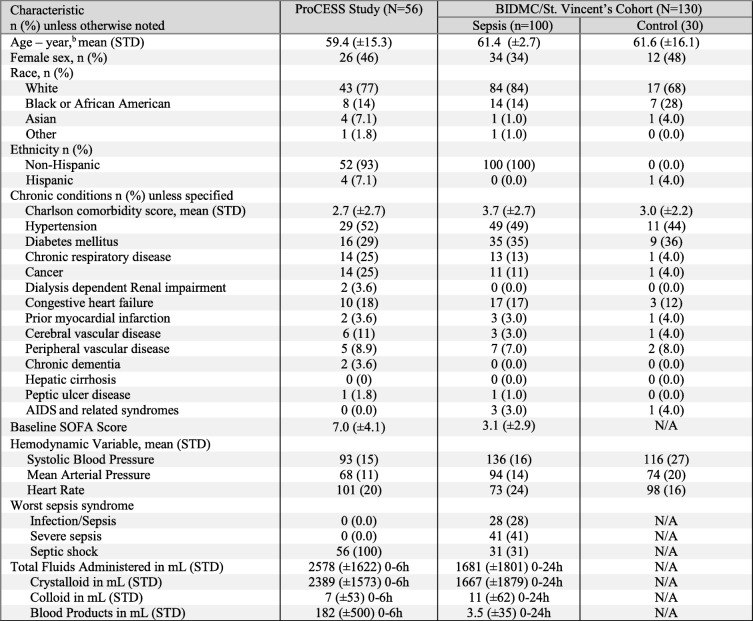
Characteristics of two sepsis cohorts

Given the known importance of the endothelial glycocalyx to vascular homeostasis, we compared plasma heparan sulfate concentrations with circulating markers of endothelial injury, coagulation, and inflammation (6 h after study enrollment). As detailed in Table 2, plasma heparan sulfate was significantly associated with the endothelial activation marker sFLT-1 (with a non-significant trend towards association with angiopoietin-2), the inflammatory marker interleukin-6, and the endogenous thrombolytic tPA. No associations were seen between plasma heparan sulfate and lactate, tumor necrosis factor-α, or D-dimer 6 h after study enrollment.

**Table 2:** Associations of circulating heparan sulfate with plasma indices of endothelial injury, coagulation, and inflammation (ProCESS cohort, 6 h after enrollment)

Marker	Pearson r	*p*-value	Number of subjects analyzed
Endothelial Injury/Activation			
sFLT-1 (soluble VEGF-receptor 1)	0.4097	0.0017	56
Angiopoietin-2	0.2367	0.0790	56
Inflammation			
Tumor necrosis factor-α	0.2325	0.1384	42
Interleukin-6	0.3537	0.0216	42
Coagulation			
Tissue plasminogen activator	0.3356	0.0114	56
D-dimer	0.0926	0.5699	40
Thrombomodulin	0.4997	<0.0001	56
Other			
Lactate	-0.0765	0.6527	37

Interestingly, we observed no association in the ProCESS cohort between plasma heparan sulfate and ANP (Fig. 1f), a hypothesized mediator of fluid-induced glycocalyx shedding [13]. Surprisingly, ANP levels 6 h after enrollment were elevated in patients who went on to survive septic shock (47.1 ± 5.3 pg/ml, *n* = 33), as compared to those who later died during their hospitalization (27.0 ± 7.8 pg/ml, *n* = 8, *p* = 0.02 by Mann-Whitney, area under ROC curve 0.7749). This lack of an association with increased mortality suggests that ANP is not a primary mediator of organ-injurious glycocalyx degradation. We observed no association between IL-6 and ANP (*r* = 0.09; *p* = 0.63, *n* = 29), contrasting previous literature implicating IL-6-mediated inflammation (and not volume overload) as the primary trigger for ANP release [33]. Finally, there was no association between brain natriuretic peptide (BNP, a natriuretic peptide also associated with fluid overload) and heparan sulfate (*p* = 0.367, *r* = 0.148, *n* = 39).

Using multivariable linear regression, we observed that plasma heparan sulfate at the end of sepsis resuscitation (6 h after enrollment) was associated with the volume of intravenous fluids administered during that resuscitation period, even when adjusting for age and severity of illness (Table 3).

**Table 3:** Heparan sulfate shedding 6 h after ProCESS enrollment is independently associated with the volume of fluid resuscitation received over those 6 h (*n* = 56)

Plasma heparan sulfate (measured at 6 hours after study enrollment)			
Variable	Parameter estimate	Standard error	*p*
Intercept	-132.27	276.3	0.63
SOFA	25.28	14.65	0.09
Age	1.53	4.04	0.71
Cumulative intravenous fluids, 0-6h	0.08	0.04	0.047

### Beth Israel Deaconess Medical Center/St. Vincent’s cohort

**Fig. 2:**
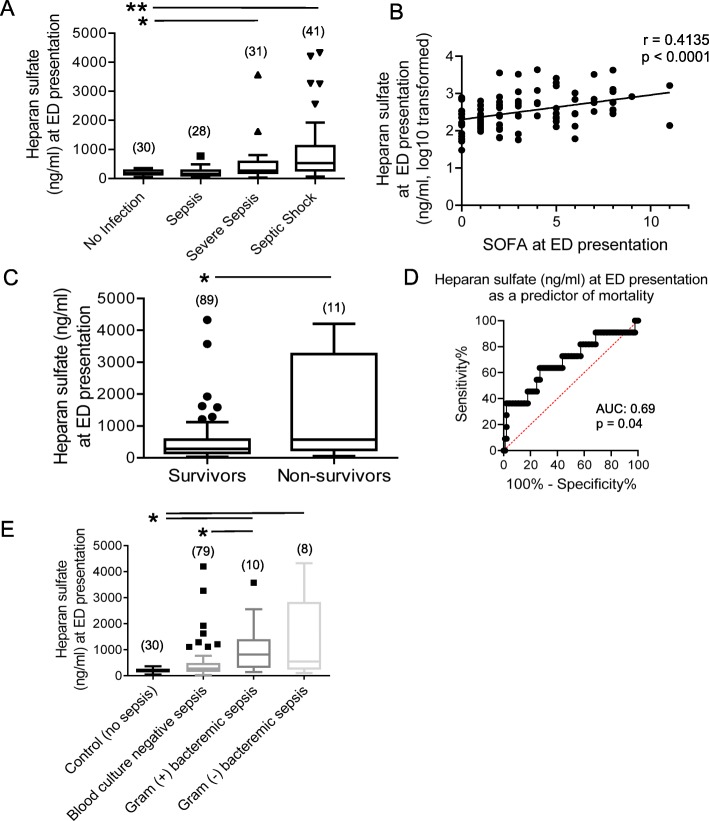
Circulating glycocalyx degradation products predict clinically relevant outcomes in sepsis patients presenting to the Beth Israel Deaconess Medical Center or St. Vincent’s Hospital Emergency Departments. **a** Elevated levels of circulating heparan sulfate at emergency department (ED) presentation were associated with a diagnosis of severe sepsis or septic shock within the ensuing 72 h (*n* = 100). **b** Heparan sulfate levels correlated with increased severity of illness (SOFA) at the time of ED presentation (*n* = 100). **c** Measures of circulating heparan sulfate in septic patients at ED presentation (*n* = 100) were significantly associated with mortality. **d** Receiver operating characteristic (ROC) curve for plasma heparan sulfate (at ED presentation) as a predictor of later in-hospital mortality. **e** Heparan sulfate plasma concentrations at ED presentation were elevated in septic patients with positive bacterial blood cultures. Three blood samples that grew *Staph. epidermidis* (contaminant) were excluded. *p < 0.05; **p < 0.0001. Parentheses represent number of patients

We analyzed plasma collected from an independent cohort of 100 septic patients (defined by Sepsis-2 criteria) at the time of presentation to the EDs of the BIDMC and St. Vincent’s Hospital (“BIDMC/St. Vincent’s cohort”, Table 1) to confirm the generalizability of these initial findings beyond our sampling of ProCESS patients. In addition, 30 non-infected ED patients served as controls. In keeping with the ProCESS cohort, we found an elevation in circulating heparan sulfate levels (at time of ED arrival) in patients who were diagnosed with severe sepsis or septic shock within 72 h (Fig. 2a). In this cohort, circulating heparan sulfate concentrations at ED presentation were significantly associated with severity of illness (SOFA) at that time (*r* = 0.4135, *p* < 0.0001, Fig. 2b). Plasma heparan sulfate at ED arrival was significantly higher in non-survivors, as compared to survivors (*p* < 0.05, Fig. 2c, d). Intriguingly, heparan sulfate shedding was more predominant in septic patients with positive blood cultures (Fig. 2e) than blood culture-negative sepsis; no differences were seen between gram positive or gram negative bacteremia.

Ninety-seven of the 100 patients in the BIDMC/St. Vincent’s cohort had serial blood draws collected at 0 h and 24 h available for analysis. In these patients, the volume of intravenous fluids administered over the first 24 h of the study correlated with the change in plasma heparan sulfate across that time period, adjusted for age and baseline SOFA (Table 4).

**Table 4:** In the Beth Israel Deaconess Medical Center/St. Vincent’s cohort (100 patients), the 24-h increase in circulating heparan sulfate (an index of ongoing glycocalyx degradation) is independently associated with the volume of fluid resuscitation received over those 24 h

Change in plasma heparan sulfate (from time of study entry to 24 hours after enrollment)			
Variable	Parameter estimate	Standard error	*p*
Intercept	-340.19	276.91	0.22
SOFA	-13.44	23.15	0.56
Age	6.50	4.26	0.13
Cumulative intravenous fluids, 0-24h	0.08	0.03	0.02

**Fig. 3:**
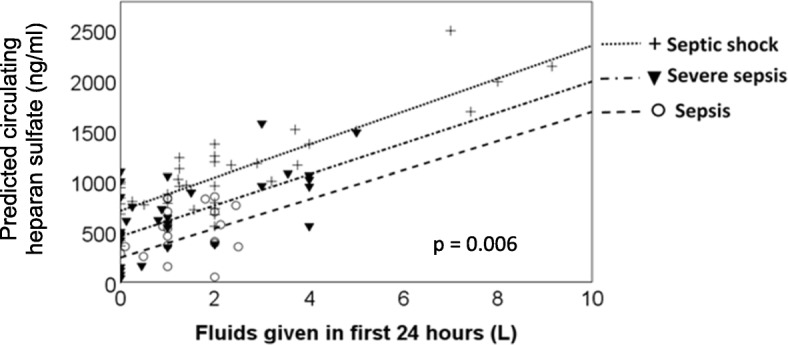
Volume of intravenous fluids administered early in sepsis predicts degree of glycocalyx degradation. The total volume of intravenous fluids administered in the first 24 h after ED presentation predicted the change in plasma heparan sulfate levels from initial blood draw to 24 h blood draw. Model *R*^2^ = 0.149, fit lines are shown for patients experiencing sepsis, severe sepsis, and septic shock, adjusted for age and baseline SOFA score

We used paired data from the BIDMC/St. Vincent’s sepsis cohort to model the 24-h change in plasma heparan sulfate from intravenous fluid volume administered across that time period, adjusted for age, baseline SOFA score, and 72 h sepsis syndrome severity. Predicted values are shown in Fig. 3, demonstrating that independent of disease severity and age, each 1 l of fluids administered was associated with a 200 ng/ml increase in circulating heparan sulfate (*p* = 0.006).

## Discussion

Our report supports preclinical observations [13] that both sepsis severity and the volume of intravenous fluids administered during sepsis resuscitation are associated with glycocalyx degradation. While our study design is unable to prove causality, the associations are consistent across patient cohorts. An injurious effect of fluid resuscitation on the endothelial glycocalyx would be expected to worsen sepsis outcomes, given the importance of glycocalyx integrity to vascular homeostasis. In healthy vessels, the intact endothelial glycocalyx functions to oppose transvascular fluid flux and leukocyte adhesion. Accordingly, pathologic loss of glycocalyx integrity during sepsis might directly contribute to the tissue edema characteristic of septic organ injury [14]. Furthermore, as the glycocalyx plays a critical role in regulating nitric oxide synthesis, septic glycocalyx degradation may contribute to the microvascular heterogeneity and vasoplegia characteristic of septic shock [13]. Exacerbation of sepsis-induced glycocalyx degradation by intravenous fluids would therefore be expected to worsen inflammatory organ injury and microcirculatory dysfunction, significantly impacting patient outcomes.

The mechanisms by which intravenous fluids could induce glycocalyx degradation are uncertain. Pre-clinical animal and human studies have shown that ANP degrades the endothelial glycocalyx [19–21], suggesting that fluid resuscitation may cause iatrogenic glycocalyx degradation in septic patients via the induction of volume overload. While our study does support an association between the volume of fluid resuscitation and glycocalyx degradation, we did not observe an association between plasma ANP (or BNP) and heparan sulfate. These findings, while observational and limited to plasma samples available from ProCESS cohort, do not support the hypothesis that natriuretic peptide-mediated degradation in response to volume overload is the primary mechanism responsible for intravenous fluid-associated glycocalyx degradation. Rather, intravenous fluids may be capable of directly inducing endothelial injury and endothelial shedding independently of fluid balance. One possible mechanism is fluctuations in endothelial shear stress caused by fluids. Sudden vascular stretch from fluid boluses paired with the presence of inflammatory mediators may stimulate endothelial expression of glycocalyx-shedding matrix metalloproteinases [34]. Furthermore, oscillatory shear stress may promote cathepsin L activation, an enzyme implicated in post-translational activation of endothelial heparanase [35]. Alternatively, isotonic fluid administration could directly activate circulating leukocytes [36, 37], potentially inducing neutrophil elastase-mediated glycocalyx degradation [38]. These speculative mechanisms will require additional translational investigation.

Intriguingly, the ProCESS and BIDMC/St. Vincent’s cohorts relied upon intravenous saline as the primary volume resuscitation agent, accounting for greater than 90% of the fluid administered in both cohorts. Emerging clinical and preclinical studies suggest that saline may be injurious when compared to balanced crystalloids [39], albumin [40], and fresh frozen plasma [41] in the resuscitation of critical illness. Future human studies will be necessary to determine if these resuscitation agents are differentially associated with glycocalyx degradation.

Our study has several strengths, including the use of two independent patient cohorts. Additionally, our findings are based upon state-of-the-art mass spectrometry analyses (LC-MS/MS MRM) of plasma heparan sulfate [28], an approach that is highly sensitive to both septic and non-septic endothelial glycocalyx degradation [31]. Circulating heparan sulfate is also highly specific to endothelial glycocalyx degradation: while heparan sulfate exists external to the vascular lumen (e.g. in the basement membrane and interstitium), negatively-charged fragments produced by degradation of extravascular heparan sulfates would be repelled by the negative charge of an intact glycocalyx, preventing plasma penetration. As such, the presence of circulating heparan sulfate fragments necessitates a breach of glycocalyx integrity. The validity of mass spectrometry measures of heparan sulfate as an index of glycocalyx degradation in our cohort is supported by the observed correlation with shed syndecan-1 (Fig. 1d), a commonly-used ELISA-based assay of glycocalyx degradation [18, 42], as well as shed thrombomodulin (Fig. 1e), an endothelial-surface chondroitin sulfate proteoglycan.

Our study also has several limitations. First, we only included a convenience sample of patients in the study cohorts and it is possible that this introduced a selection bias. Second, despite our study being amongst the largest to employ mass spectrometry to investigate glycocalyx degradation in humans, the overall sample size and number of deaths is still relatively low; thus, larger studies are needed to validate our findings. Third, it is possible that other measures of glycocalyx degradation are needed above and beyond circulating levels of heparan sulfate to have a comprehensive readout of glycocalyx degradation. Finally, it is important to emphasize that despite our use of multivariable modeling to account for measured confounders, our cohorts were underpowered to address additional pertinent variables that could affect glycocalyx integrity, such as underlying comorbidities [43], use of stress-dose glucocorticoids [44], and appropriate antibiotic choices (Fig. 2e). Our observational study is therefore unable to exclude the contribution of these and other unrecognized confounders of the observed association between fluid resuscitation and glycocalyx degradation. As such, we are unable to conclude causality.

Our observational findings therefore require confirmation by prospective, randomized studies. The Prevention and Treatment of Acute Lung Injury (PETAL) Network has recently initiated a large study of volume resuscitation practices in sepsis. This Crystalloid Liberal or Vasopressors Early Resuscitation in Sepsis (CLOVERS) study will compare the use of a liberal fluids protocol (larger volume of fluids prior to the initiation of vasopressors) with a restrictive fluids protocol (smaller amounts of intravenous fluids and early use of vasopressors) in patients with sepsis-induced hypotension [45]. Studies such as CLOVERS will provide opportunities to leverage randomized assignment of resuscitation strategies, allowing for better insight into the causal relationship between intravenous fluids and glycocalyx degradation.

## Conclusions

Our report demonstrates an association between fluid resuscitation and glycocalyx degradation in sepsis, supporting observations made using preclinical models of endotoxemia [13]. Future randomized controlled studies will provide an opportunity to confirm a causal association.

## Data Availability

The datasets generated and/or analyzed during the current study are available from the corresponding author on reasonable request.

## Abbreviations

ANP: Atrial natriuretic protein
BIDMC: Beth Israel Deaconness Medical Center
BNP: Brain natriuretic peptide
CLOVERS: Crystalloid Liberal or Vasopressors Early Resuscitation in Sepsis
ED: Emergency department
LC-MS/MS MRM: Liquid chromatography-mass spectrometry multiple reaction monitoring
PETAL: Prevention and Treatment of Acute Lung Injury
ProCESS: Protocolized Care for Early Septic Shock
sFLT-1: Soluble fms-like tyrosine kinase-1
SIRS: Systemic inflammatory response syndrome
SOFA: Sequential Organ Failure Assessment
tPA: Tissue plasminogen activator
